# From consent to participatory governance: The need for multi-stakeholder alignment in iPSC-based drug repurposing

**DOI:** 10.1016/j.stemcr.2026.103062

**Published:** 2026-09-03

**Authors:** Esther A.M. Bührman, Alain Geille, Martina C. Cornel, Wouter P.C. Boon, Vivi M. Heine, Tessel Rigter, SIMPATHIC Consortium

**Affiliations:** 1 Section Community Genetics, Department of Human Genetics, Amsterdam Public Health Research Institute, Personalized Medicine, Amsterdam UMC, Vrije Universiteit Amsterdam, 1105 AZ Amsterdam, the Netherlands; 2 Euro-DyMA, Myotonic Dystrophy Association, Paris, France; 3 Copernicus Institute of Sustainable Development, Utrecht University, Princetonlaan 8a, 3584 CS Utrecht, the Netherlands; 4 Department of Child and Adolescent Psychiatry, Emma Center for Personalized Medicine, Emma Children’s Hospital, Amsterdam UMC, Amsterdam Neuroscience, Amsterdam, the Netherlands; 5 Department of Complex Trait Genetics, Center for Neurogenomics and Cognitive Research, Amsterdam Neuroscience, Vrije Universiteit, Amsterdam, the Netherlands

**Keywords:** iPSC, drug repurposing, governance, rare disease, neurological, patient engagement, participatory, multi-stakeholder

## Abstract

Induced pluripotent stem cells (iPSCs) are increasingly used as disease models to accelerate drug repurposing, especially for rare diseases. While ethical and regulatory issues in iPSC research have been widely discussed, little is known about how these challenges are addressed in practice. To provide insights into the current practice, challenges, and opportunities in the governance of research on iPSC-based drug repurposing for rare neurological disorders, semi-structured interviews were conducted with various experts from eight countries in the context of the SIMPATHIC project. The results indicate that this research context requires improved information provision for minors and individuals with cognitive impairment and more clarity regarding commercial use and reporting of findings. Uncertainty in governance procedures hinders collaboration, validation, and clinical translation in iPSC-based drug repurposing. Responsible (re)use of iPSCs requires dynamic, transparent, and participatory governance structures based on shared decision-making with all stakeholders involved.

## Highlights

• Current consent models do not meet stakeholder needs in iPSC-based drug repurposing
• Ambiguous governance hinders collaboration, validation, and clinical translation
• Multi-stakeholder alignment is key for international exchange and collaboration
• iPSC-based drug repurposing requires dynamic and participatory governance

## Teaser

In this article, Bührman and colleagues provide insight into the current practice, challenges, and opportunities in iPSC-based drug repurposing based on a qualitative interview study with various stakeholders. The authors propose key recommendations for co-creating dynamic, transparent, and participatory governance structures in iPSC research for rare diseases.

## Introduction

Induced pluripotent stem cell (iPSC) technology has transformed biomedical research by enabling patient-specific disease modeling and drug screening *in vitro* for a wide range of diseases (Bartfeld and Clevers, 2017; Cerneckis et al., 2024). For rare diseases, patient-derived iPSCs are particularly valuable because they carry the individual’s disease-specific genetic variations and can reduce reliance on animal testing. This is important as for some disorders, no suitable animal models exist, or available models fail to accurately capture the underlying genetic features or clinical manifestations, limiting their predictive value (Farkhondeh et al., 2019; Lorenz et al., 2017; Sayed et al., 2016). As a result, patient-derived iPSCs are increasingly used in drug repurposing projects to accelerate treatment development for patients with high unmet medical needs (Paquot et al., 2024; Roessler et al., 2021). For example, the SIMPATHIC project (accelerating drug repurposing for rare neurological, neurometabolic, and neuromuscular disorders by exploiting similarities in clinical and molecular pathology) uses iPSC-derived disease models for drug screening (van Karnebeek et al., 2025), illustrating a specific context in which iPSC-based drug repurposing can contribute to faster treatment development for patients with a rare neurological disease.

Although promising, iPSC-based drug repurposing for rare neurological disorders raises distinct research governance challenges. Following Morrison et al. (2015), we understand *governance* as encompassing all formal and informal methods of coordination and organization. Most governance challenges in iPSC research discussed previously concern consent procedures, data sharing, and commercialization (Hyun et al., 2020; Moradi et al., 2019; Pichl et al., 2023). For drug repurposing in rare neurological disorders, there might be additional research governance challenges as it involves long-term cell storage, data sharing across jurisdictions, and engagement with small and often vulnerable patient populations. In this study, we focus on three governance challenges in iPSC research: choosing appropriate consent models, organizing (inter)national exchange and collaboration, and ensuring meaningful patient engagement.

First, most of the ethical discussion on the governance of iPSC research has revolved around consent. Under appropriate conditions, cell lines can be stored indefinitely, while future uses of iPSCs are difficult to predict (Kataoka et al., 2024; Pichl et al., 2023). This raises the question whether patients have given informed consent for each conceivable application, including ethically sensitive ones such as neuronal organoids and commercial use. For rare neurological disorders, additional complexities arise because patients are often minors and/or legally incompetent. They require a legal guardian to give consent for storing their cells until they reach the age and maturity at which they can decide for themselves, which makes (re)consent a complex issue (Kranendonk et al., 2017). In these cases, patients may be able to provide *assent*, which is the affirmative agreement to participate in accordance with their capacities. (N.B. *assent* does not replace a legally valid *consent*.) Another practical complexity is the possibility for patients to withdraw, for example, if cells are already transferred to other organizations (Lewis and Holm, 2022).

The new use and purpose of previously stored cell lines call for a critical re-evaluation of the consent models discussed in literature (de Jongh et al., 2022). Mikkelsen et al. (2019) argue that broad consent, widely used in biobanking, should meet specific criteria to safeguard participant autonomy, such as continuous information and ethical review. They propose the *information*, *value*, and *duration* criteria to evaluate consent models. In addition, Barnes et al. (2025) advocate a demonstrated consent approach, which should also incorporate the ethical principles of justice, beneficence, and non-maleficence for individuals and society (the *balance* criterion). The issues described earlier, plus the fact that ethical considerations and social values are subject to change, suggest a need for a more dynamic consent model than is currently common practice, enabling patients to regularly receive updates and change preferences regarding the use of their cell lines (Boers and Bredenoord, 2018; Isasi et al., 2024; Kaye et al., 2015; Prictor et al., 2020). Yet, it remains unclear how such consent models are implemented in practice within translational, multi-country iPSC-based drug repurposing initiatives.

Second, a recently evolving governance challenge in iPSC research concerns the organization of international exchange and collaboration. Effective iPSC-based drug repurposing often requires cross-border exchange of cell lines and collaboration between public and commercial partners. In the rare disease field, accurate disease models are scarce, and international collaboration is needed to make optimal use of the existing knowledge and expertise. These collaborations must navigate national and local regulations (López et al., 2016; Monaco et al., 2014). These regulations can differ substantially, particularly for biobanking and the use of bodily material (Bentzen, 2024; Kaye et al., 2018; López et al., 2016; Morrison et al., 2015; Zika et al., 2011). Historical iPSC collections may also have been obtained through different consent procedures, even within the same country. International research collaboration is thus subject to regulatory fragmentation, which could delay the research and translation of iPSC-based discoveries into clinical applications.

Third, achieving meaningful involvement of patients in iPSC research poses a governance challenge. There has been increased attention for a more active role of research subjects in iPSC research (Kaye et al., 2018; Lensink et al., 2022). For example, a study involving patients and parents of patients with cystic fibrosis indicates that long-term patient engagement is essential for sound governance of organoid technology (Boers et al., 2018). Rare disease patients introduce specific considerations, as they represent a small and vulnerable population with limited time and resources. At the same time, due to high unmet medical needs, patient organizations have historically been very active in this field, for example, in the support of rare disease patient registries (López et al., 2016), facilitating international research collaboration and clinical trials (Boon and Broekgaarden, 2010). Although patient engagement is considered important for developing appropriate governance models, it is unclear how patients and their representatives are currently involved in iPSC-based drug repurposing projects.

In summary, the differences in consent models, national and local governance procedures, and the levels of patient engagement complicate the governance of iPSC research. It is currently unclear (1) how the governance of iPSC research is organized in practice and (2) which challenges and opportunities stakeholders perceive for iPSC-based drug repurposing, particularly for rare neurological disorders. Using the context of the SIMPATHIC project as a research case, this study seeks to provide insight into the current governance practice and aims to define key recommendations to facilitate the governance of international iPSC research in the context of rare diseases.

## Results

### Interview participants

In total, 17 participants were interviewed from the Netherlands (*n* = 7), Germany (*n* = 3), Luxembourg (*n* = 1), Greece (*n* = 1), the United Kingdom (*n* = 2), Finland (*n* = 1), Australia (*n* = 1), Italy, and Sweden (*n* = 1). The sample included 5 iPSC researchers, 2 clinicians, 3 patient experts, 3 ethical experts, 2 data experts, and 2 legal experts (Table 1). Details about the empirical approach can be found in supplemental information S2 and S3.

**Table 1:** The number of interview participants (R) with their expertise and type of organization

#	Expertise	Type of organization
R1	iPSC researcher	University Medical Center
R2	iPSC researcher	university
R3	iPSC researcher	University Medical Center
R4	patient expert	patient organization
R5	patient expert	patient organization
R6	iPSC researcher and clinician	University Medical Center
R7	ethical expert	University Medical Center
R8	legal expert	university
R9	ethical expert	university
R10	iPSC researcher	University Medical Center
R11	ethical expert	university
R12	clinician	University Medical Center
R13	data expert	University Medical Center
R14	patient expert	patient organization
R15	data expert	University Medical Center
R16	iPSC researcher	University Medical Center
R17	legal expert	university

In the interviews, several challenges and opportunities were identified for iPSC research in the context of drug repurposing for rare neurological disorders. The themes are structured around the three governance issues as presented in the introduction: choosing appropriate consent models, organizing international exchange and collaboration, and patient engagement.

### Choosing appropriate consent models

The opinions of interview participants on the best type of consent differed, including specific consent (R5 and R14), broad consent (R13, R15, and R17), and dynamic consent (R11). (Dynamic consent was defined as a consent model that involves ongoing communication about the use of the iPSCs, including updates when new studies are initiated and possibilities for reconsent.) The identified challenges and opportunities clustered around the four criteria for consent in biobank research (information, value, duration, and balance criterion) as defined by Mikkelsen et al. (2019) and Barnes et al. (2025) (Table 2). In the following section, we describe the themes per consent criterion.

**Table 2:** An overview of the criteria for consent in biobank research used in this study

Consent criterion	Description
Information criterion	“Inform potential participants about the relevant risks and benefits of biobank research (especially informational risk) so that [they] can make an autonomous choice about participation in light of this information.”
Value criterion	“Offer participants the opportunity to assess whether the research to be conducted with biobank materials is in line with their personal values, and to make consent decisions based on this.”
Duration criterion	“Afford ethical protection for the duration of the subject’s participation in the biobank and give the subject a real and meaningful opportunity to reassess [their] consent for that duration and an actionable right of withdrawal (provided, of course, that the participant is still alive and competent).”
Balance criterion	“Balance the protection of individual autonomy with wider societal interests in the progress of science and medicine, while also honoring the demands of justice.”

The description of the information, value, and duration criterion is cited from Mikkelsen et al. (2019), and the description of the balance criterion is cited from Barnes et al. (2025).

#### Information criterion

The information criterion states that potential participants should be informed about the relevant risks and benefits of biobank research so that they can make an autonomous choice (Mikkelsen et al., 2019). Interview participants differed in their opinions on what kind of information should be provided in the consent procedure. Overall, patient experts and ethical experts felt that the specific purpose of the research should be clearly explained, while iPSC researchers and data experts expressed concerns that this would hinder follow-on research, because participants would then not have consented to these research projects. Therefore, a data expert (R13) recommended not specifying the disease area in consent. Patient experts (R4 and R5) proposed minimum requirements for consent: (1) an overview of risks and benefits, (2) information on the purpose of the study or project, (3) information on the protocol and invasiveness, (4) information on how participants will be informed about the results, and (5) details on storage and sharing with academic and/or commercial organizations. The latter was considered especially important in drug repurposing, because exchange with commercial organizations may be more likely than in other types of (basic) iPSC research.

Interview participants also argued for more tailored information. Patient experts stressed that information needs differ per individual and should be captured in the consent process, for example, preferences about receiving research findings (R5). Plain language should be used, and written information in a consent document was considered insufficient to fully explain all risks and benefits, especially for patients with rare neurological disorders who may have cognitive impairment. Multiple interview participants (R6, R8, and R11) advocated shorter texts and additional visualizations, videos, presentations, or other sources about iPSC research to improve the consent procedure. As an example of best practice, a patient expert (R5) described that “ethics committees would involve patient groups to make sure […] it’s clear language, it’s understood by the patients, which can be a challenge sometimes with people with cognitive issues.”

The specific area of research also matters: interview participants felt that the personalized aspects and potential medical implications of drug repurposing research make it more important to carefully consider how communication with patients should be organized. For example, once you start selecting which patients or which specific mutations a treatment might work for, it is important that this information reaches both clinicians and patient communities, as it affects clinical trial eligibility. However, an iPSC researcher (R16) indicated uncertainty about who is responsible for reporting these findings in industry collaborations: “The information on what type of mutations that treatment is going to work is then very important, but in that case, such a company should of course use that information for the clinical trials. And then I don’t know whether we would be the ones to share that, or whether the company would have to do that.”

In the case of incidental health findings, some interview participants (R10, R15, and R16) found it unclear whether and how they should be reported. They found it important that the policy on reporting incidental findings is determined in advance and that the procedure is included in the consent form. The lack of clear national and international policies on how to deal with such findings makes it difficult to align these practices, as explained by a data expert (R15): “We don’t even have a policy in the Netherlands on how to deal with incidental findings when it comes to scientific research, let alone European policy.” The question of what to share is even more important given the uncertainty around future research and technologies, making it difficult to predict what kind of findings might be relevant.

To summarize, patient experts advised tailoring the information to the needs and preferences of the patient. For drug repurposing, interview participants attached particular importance to information about commercial use and the reporting of (incidental) health findings.

#### Value criterion

The value criterion states that participants should be able to assess whether the research is in line with their personal values and to make consent decisions based on this (Mikkelsen et al., 2019). (This goes beyond providing information about the risks and benefits of the research, as discussed in the information criterion. The value criterion states that underlying values of participants should be addressed in the consent procedure.) Interview participants agreed that, at a minimum, the research purpose and use of cells must be clear for meaningful value-based decision-making. Most interview participants (R1, R2, R3, R6, R7, R11, R13, and R14) experienced that patients were generally positive toward participation in iPSC research and mentioned no specific concerns or restrictions regarding drug repurposing. They reported that most of the time, patients are aware that the research would likely not result in a personal clinical benefit and that they participate based on altruistic motivations. However, a patient expert (R4) added as a requirement that it is important for patients that the use of their cells is in line with their ethical considerations, for example, regarding privacy and commercial use. Multiple interview participants (R3, R5, R8, R9, R11, and R14) also underlined trust as a prerequisite for participation, for example, in terms of transparent communication and the clinician-patient relationship.

For iPSC-based drug repurposing, patient cell lines often need to be exchanged with commercial parties, for instance, to outsource drug screening. Data experts and iPSC researchers indicated variations and unclarity in local policies regarding commercial use. New consent forms increasingly specify whether cell lines may be exchanged with commercial parties, but this is not always the case for cells that have been obtained earlier. Moreover, it is unclear if collaboration with industry for drug screening falls under commercial exploitation, as explained by a data expert (R13): “So any kind of drug screening is really hard. Drug screening is a real gray zone. It’s a real gray zone in terms of research and commercialization.” This unclarity is also experienced by an iPSC researcher (R16): “It is indeed a vague boundary, what exactly they [biobanks] consider as commercial. And what they say varies from company to company. Of course, commercial is also a term that can be defined very broadly. All the work done at the university, is that okay? Or is it the moment you collaborate with industry that pays for it … is that allowed or not?” To illustrate this ambiguity, the two aforementioned experts had different definitions of commercialization based on their experience. The data expert stated that “an outsourced assay for screening is commercial exploitation,” whereas the iPSC researcher believed that “they really mean that you use them [iPSC lines] in a commercial way, for example, that you are going to sell them as a company or make cell products from them.” These different views show the difficulty for participants to assess whether commercial use of cell lines aligns with their values.

Several interview participants (R4, R5, R8, R9, and R11) also expressed concerns regarding intellectual property rights obtained or excessive profits being made by companies. Most interview participants felt that large commercial profits should be avoided. A legal expert (R8) states: “The non-commerciality principle, especially in the case of bodily material, will have to be paramount,” meaning that it should not be a source of financial gain for either patients or researchers. A patient expert (R5) stressed the need for transparency about the use of the cell lines in and outside the university, and the potential profits: “And the biggest issue around that [transfer/exchange] has been [that] it depends on what happens with those samples. So, if they’re used by university researchers for basic research, then that’s one thing. If those samples are sold in five or ten years’ time to a giant pharmaceutical company to make a profit, then that’s a different thing altogether, ethically, and that needs to be reflected on the consent form.”

Overall, a trust relationship between patients and clinicians and information and transparency about commercial use and profits were considered key factors to facilitate value-based decision-making.

#### Duration criterion

The duration criterion states that there should be ethical protection for the duration of the subject’s participation in the biobank, with a meaningful opportunity to reassess consent and a right to withdraw (Mikkelsen et al., 2019). Several interview participants (R1, R2, R3, R7, R8, R9, R10, and R11) felt that reconsent for the use of existing cell lines may be necessary over time, for example, in case of new technological developments or when exchange with (commercial) parties was not included in the original consent. Some interview participants (R6, R7, R10, and R10) also recognized the need for reconsent when participants who were minors at the time of donation reach a legally competent age. None of the interview participants were aware of standard procedures to ensure reconsent.

At the same time, some interview participants (R1, R3, R8, R10, R13, and R15) considered recontacting donors as practically challenging and risky, for example, because they were afraid that they might decide to withdraw from participation. Some researchers were also uncertain about the best way of recontacting participants, because they were not sure how to reach individual patients (as they did not have access to the contact information) or whether patients would appreciate being recontacted. There was also uncertainty regarding the procedure for deceased patients, which is particularly relevant in rare neurological disorders with limited life expectancy. This confusion was described by an iPSC researcher (R2): “For those severe diseases, you know, with low life expectancy, it just might even be more dramatic. Because if you have a cause of disease with a life expectancy of four years and you get the iPSCs in the age of two, then you basically after two years … Um. Yeah.” A data expert (R13) explains that recontacting patients is therefore not always an option: “In some cases, the donor is unfortunately deceased, so that is not an option. Unfortunately, sometimes the individual might be very ill, you know, it’s just not an option.”

In summary, multiple interview participants found reconsent important for new research applications or when patients reach the age of legal capacity. However, interview participants also discussed practical challenges for recontacting patients, such as the risk of withdrawal and problems with reaching patients.

#### Balance criterion

The balance criterion is about balancing the protection of individual autonomy with wider societal interests in the progress of science and medicine, while also respecting justice (Barnes et al., 2025). Several themes were identified that affect this balance for iPSC-based drug repurposing.

For scientific progress, insufficient quality control was often reported as obstructing the validation and reproducibility of results in iPSC research. Interview participants (R9, R13, and R16) indicated that iPSC research suffers from a lack of characterization, i.e., performing specific analysis to confirm the quality of cell lines. For drug repurposing research potentially resulting in a clinical trial and eventually treatment (re-)development, the characterization of the cell lines is even more important. This is illustrated by an experience of an iPSC researcher (R16), who shared that they discovered a mutation in a patient cell line in a different location than indicated in the data. Even though they managed to report this finding to the original researcher and clinician, they were unsure whether that information reached the patient. This was seen as a significant problem, especially if that patient receives a specific treatment unlikely to be effective with this mutation. Thus, incorrect characterization can have undesired consequences, not only for the researchers themselves but also for the clinical translation of iPSC-based drug repurposing. Interview participants mentioned insufficient time and resources and the absence of mandatory checks as reasons for the lack of characterization. As a solution, interview participants encouraged the publication of protocols and lab resource articles (R1, R2, R12, R13, and R16) and educating early career researchers (R9) about quality control.

Another relevant factor for the balance criterion mentioned by interview participants is the potential of iPSC-based drug repurposing to reduce the need for animal testing and thereby support the three Rs of reduction, refinement, and replacement (European Commission, 2025). As an iPSC researcher (R10) explains: “Repurposing makes it all a bit easier. You need to do much less, perhaps even no animal testing at all.” However, ethical experts (R7 and R9) questioned this “promise” of animal-free research. One ethical expert wondered if the reduction of preclinical animal studies would increase the burden or risks for patients: “You know, I’m genuinely worried that some of these things with using less animals could go in the direction of using more humans, […] including humans who are vulnerable.” They emphasized the importance of a safe translation from preclinical to clinical research in drug repurposing.

Concerns about clinical translation in iPSC-based drug repurposing were shared by other interview participants (R3, R4, R5, R7, R9, R10, R16, and R17), especially focusing on the risk-benefit balance for rare disease patients in clinical trials. The benefits should outweigh the risks related to privacy, safety aspects, and burden for the patient. They found it important that the preclinical evidence on safety is maximally safeguarded to prevent harm to vulnerable populations. Interview participants distinguished between benefits for an individual patient, benefits for the patient community, and commercial benefits. The potential collaboration with industry in drug repurposing raised the question who will benefit from the research. Ethical and patient experts also recommended early consideration of the implementation and access to repurposed drugs, as explained by R7: “And actually from an ethical perspective this is also necessary, because otherwise we are [...] investing in research that ultimately may not translate into knowledge that we can use as a society and in that case we are not doing it right, also not for the people who participate.” In other words, it was found important that the iPSC research would eventually benefit rare disease patients.

In sum, interview participants highlighted scientific quality (especially cell line characterization), reduction of animal testing, and safe clinical translation as core components of the balance criterion in iPSC-based drug repurposing. Interview participants emphasized the importance of preventing harm in iPSC research and maximizing the benefits for rare disease patients.

### Organizing exchange and collaboration

(International) exchange and collaboration are considered important aspects for iPSC-based drug repurposing. Such collaborations are even more important for research into rare diseases, where individual research centers often only have direct access to a limited number of patients with the same diagnosis. Interview participants described several challenges in organizing the exchange of cell lines, related to registration, transfer, regulatory differences, and multi-stakeholder interaction.

#### Registration of cell lines

Interview participants (R1, R2, R3, R9, R10, R12, and R13) reported a lack of incentives for the registration of cell lines, due to unclear procedures, difficulties with the registration process, and different local requirements. Yet registration was considered crucial for collaboration, enabling other researchers to find and access data, obtain cell lines, and validate findings. To increase accessibility, interview participants mentioned solutions such as making registration a requirement for funding (R13) or publication (R1) of research. Public funding opportunities (R3) and funding by patient organizations (R4, R5, and R13) were also suggested as structures that could encourage cell line registration in the rare disease field. For example, a data expert (R13) described the following: “So interestingly now we’re working with a number of different patient organizations where they said, OK, we’re going to raise our own money, we’re going to make our own iPSC lines and we’re going to put them into a public repository and we’re doing that with a number of different groups at the moment.”

#### Transfer of cell lines

Interview participants also reported difficulties in transferring cell lines to other academic institutions, other countries (within and outside the EU), and commercial parties. The transfer of cell lines was perceived as a difficult process by several experts (R1, R2, R3, R6, R9, and R14) and as a barrier to iPSC research due to (interacting) ethical, legal, and practical restrictions (R7, R10, R12, and R13). Local ethical review boards may require specific consent for transfer, which can be difficult to obtain in practice. Interview participants particularly expressed frustration around the length and complexity of the material transfer agreement (MTA) procedure, characterizing it as a “painful process” (R1) or “a headache” (R14), especially in international collaborations.

Sometimes, limitations on the transfer of patient cell lines stemmed from the personal considerations of the iPSC researchers, because they did not want to lose control over the use of the cells (R6): “I feel like at this point I don’t want to risk it. […] Especially when we’re sequencing all of the samples with external companies, even if they are based in Europe, it’s still out of my hands.” Another reason for not sharing iPSCs was unclarity in regulations (R16): “What I’m never quite sure about is whether we’re allowed to share them with other companies as well, so we don’t, we just don’t. So, we really keep them here.” Interview participants therefore recommended explicitly addressing transfer in consent forms, potentially as a separate consent item. They also saw opportunity in standardizing MTAs and making use of larger iPSC banks and collaboration agreements in drug repurposing for rare diseases.

#### Regulatory differences and harmonization

Interview participants indicated regulatory differences between organizations and countries as a challenge for international exchange and collaboration in iPSC-based drug repurposing. Legal experts (R8 and R17) also mentioned the interaction between different legislations as complicating governance. The differences in conditions for the use of iPSCs as described in legal documents such as consent forms and MTAs were considered unclear by multiple interview participants (R6, R9, R11, R14, and R16). Thus, these documents were not always considered sufficient to ensure meaningful consent, as explained by an ethical expert (R11): “So I don’t care if in the legal document that this is mentioned in a line. I mean, people need to understand.” Regulatory differences might also result from the complexity of relevant legislation such as the General Data Protection Regulation (GDPR), leading to different interpretations by legal experts.

Apart from differences in laws and regulations, researchers and clinicians experienced differences in local procedures for working with iPSCs. They described a (perceived) lack of guidelines, resulting in self-governance and a strong responsibility on the researchers themselves for setting up research infrastructures that facilitate responsible use of iPSCs. Interview participants (R2, R6, R9, R13, and R16) advocated harmonization to streamline local governance procedures and facilitate collaboration, for example, through standardized MTA templates with custom clauses. This harmonization could also lead to a more sustainable exploitation of pioneering local initiatives, so that not every project has to “reinvent the wheel.” On the other hand, one of the interview participants (R9) questioned the feasibility and value of harmonization. Trust, transparency, and having shared interests were mentioned as key values that are most important for sound governance.

#### Multi-stakeholder interaction

The interaction between different stakeholders in shaping the governance around iPSC research was also seen as a challenge for collaboration. For example, communication between iPSC researchers and policymakers was perceived as difficult due to a lack of understanding about stem cell research and misconceptions about consent. According to a legal expert (R8), this means “that you always need translations in order to know, understand, and comprehend the law.” Furthermore, iPSC researchers explained that their dependence on legal experts for obtaining or exchanging cell lines is not ideal. A data expert (R15) reported that researchers often lack legal knowledge, while legal experts lack knowledge on the specifics of the research.

Furthermore, the division of tasks between clinicians and researchers can also complicate collaboration in iPSC research. As an iPSC researcher (R16) states: “Doctors see patients, researchers see cells. There is a gap between the two.” On the one hand, this division has advantages: it supports privacy by limiting researchers’ access to identifiable patient data and helps avoid confusion between research and care in the consent procedure (R17). On the other hand, iPSC researchers found it difficult that they did not always know the details of the consent form, including policies and preferences for reporting back findings. As a result, iPSC researchers do not always know the needs and expectations of the patients involved.

In summary, the lack of registration and difficulties with transferring cell lines were seen as barriers for organizing exchange and collaboration, further complicated by regulatory differences and challenges in multi-stakeholder interaction.

### Patient engagement

Interview participants described meaningful engagement of public stakeholders, especially patients, as central to the governance of iPSC-based drug repurposing. In the following section, we describe existing patient engagement activities and address the challenges of communication and expectation management.

#### Engagement activities

Interview participants described various forms of patient involvement, mostly focused on information provision (newsletters [R10], websites [R11], webinars [R3], and presentations [R10]) and consultation (gatherings [R2], conferences [R14], surveys [R14], and interviews [R7]). A few examples involved mutual exchange and learning between iPSC researchers and patients, such as lab visits (R1 and R2), workshops (R6 and R14), or deliberative events (R9) where participants are involved in shared decision-making about research governance. Two patient experts (R4 and R14) explained that patient organizations are sometimes invited to participate in an ethical review process and to check consent forms, but this is not standard practice. This involvement seems more common in projects involving a clinical trial, as explained by a patient expert (R14): “So we tend to get more involved in more clinical projects, but I don’t remember ever having been asked to look at consent forms for getting samples so much.” Patient organizations also engage in other governance processes, such as the MTA procedure. Furthermore, multiple interview participants (R1, R4, R5, R10, R12, R13, R14, and R16) provided examples of patient organizations that operate as funders of (drug repurposing) projects for rare diseases, where they are closely involved in the organization of iPSC biobanks and communication about the research.

#### Language and communication

Although all interview participants were actively concerned with (promoting) patient engagement in iPSC research, several challenges for patient engagement in iPSC research were identified. Two iPSC researchers (R1 and R6) and two patient experts (R4 and R14) mentioned choosing the right language as a challenge in the governance of iPSC research. The iPSC researchers both acknowledged that researchers are used to communicating with a funder’s audience and therefore could lack skills to communicate effectively with non-scientific audiences. According to various interview participants (R6, R9, and R15), professional associations should be encouraged to make greater efforts to inform a wider audience about iPSC research. It was also recommended to involve communication experts who can bridge the gap between the different stakeholders involved in iPSC research governance.

#### Expectation management

Furthermore, interview participants expressed that they find it difficult to manage expectations toward patients. An iPSC researcher (R1) became aware of the impact of communication after a misunderstanding with the family of a patient: “You need to inspire them so that they see there could be something, but at the same time be very, very realistic that for their child, probably nothing […] can be done.” According to an ethical expert (R9), expectation management is complicated by a “therapeutic misconception” about iPSC research and stem cell models often being associated with stem cell therapy. This is also experienced by a patient expert (R14), who therefore actively adjusts the language and explanation about iPSC-based drug repurposing: “And so we’re quite aware of that, when we talk about it and making sure that you don’t sort of, you kind of just say it’s a model to test drugs […] to sort of make people understand that we’re not talking about a stem cell replacement therapy or things like that.” Another aspect of expectation management that interview participants (R7 and R26) found important but often lacking is providing clarity about whether and when patients will receive research updates, e.g., in the form of interim results.

Overall, patient engagement activities are encouraged by interview participants, but in practice, patients rarely play an active role in governance. The perspectives of patients are deemed essential for choosing appropriate language and managing expectations about iPSC-based drug repurposing.

## Discussion

The aim of this study was to provide insights into the current practice, challenges, and opportunities in the governance of research on iPSC-based drug repurposing for rare neurological disorders. In this study, we focused on three governance challenges in iPSC research: choosing appropriate consent models, organizing (international) exchange and collaboration, and patient engagement. The findings show that existing consent models insufficiently address the specific needs and interests of all stakeholders in this translational field. Moreover, the uncertainty around consent forms and other governance procedures does not merely raise ethical concerns but also has practical consequences: it hampers collaboration, validation, and clinical translation in iPSC research, all of which are crucial for drug repurposing.

Both literature and practice continue to focus primarily on consent as a way to address governance ambiguities. Interview participants frequently point to more clarity in consent forms as the key solution. While this may be valuable for future consent procedures, researchers in practice often rely on previously obtained consent forms that do not account for all future practices and exchanges related to drug repurposing. Future collaborations, commercial involvement, clinically relevant findings, and opportunities for translational development often emerge after consent has been obtained. The limitations in current consent models indicate that, in addition to appropriate consent, effective governance of iPSC-based drug repurposing requires ongoing interaction and alignment among all stakeholders, allowing decisions and expectations to be revisited over time. Although efforts are made to involve patients in the governance of iPSC research, our results indicate that meaningful patient engagement practices remain fragmented and underdeveloped. In the following section, we discuss key findings and use those to define recommendations for developing dynamic, transparent, and participatory governance models to facilitate international iPSC research in the context of rare diseases.

### Choosing appropriate consent models: The limitation of current consent forms for iPSC-based drug repurposing

Previous studies show that participants’ understanding of consent forms in biobanking is often limited (Beskow and Weinfurt, 2019; McCaughey et al., 2016a), suggesting that communication on iPSC research in the consent procedure should be significantly improved. The challenges described for the information criterion indicate that the information should be tailored to disease-specific contexts. For rare neurological disorders, this is particularly important, as many participants are minors and/or individuals with cognitive impairment. Consent procedures must therefore be understandable and meaningful for these groups. This can be achieved by involving patient experts in the design and review of communication tools, as previously described for rare disease research (Gainotti et al., 2016). Information provision should also be embedded in a dialogue that allows clinicians and researchers to respond to the individual needs of patients, rather than limiting this to a static consent form. This approach has also been advocated in genomic medicine (Vears, 2025). Additional materials such as visual aids, videos, and layered information formats can further support meaningful decision-making (McCaughey et al., 2016b). Funders can play an active role in stimulating the development of communication tools that are tailored to the needs and preferences of patients.

Furthermore, interview participants reported a lack of clarity regarding commercial use and reporting of findings for iPSC-based drug repurposing. A previous study about organoid research points out that it is important that therapeutic misconceptions are prevented and that patients know what they can expect from the research in terms of (clinical) benefits (Ravn et al., 2023). Regarding the definition of commercialization, our results indicate that what is often labeled as “commercial use” in practice more commonly involves the controlled sharing of cell lines for a specific research purpose (e.g., drug screening) rather than the sale of cell lines to companies with unrestricted freedom to operate. Participants should be clearly informed about potential commercial uses, exchanges with industry partners, and the generation of profits based on their contributions (Minssen and Schovsbo, 2014). They should also know which (health) findings will be reported and within what time frame this can be expected (Muller et al., 2023). This is even more important for iPSC-based drug repurposing, as the research might have a direct impact on the life of the patients, e.g., they might benefit from drug candidates identified in the screening. The research may also yield findings that are relevant for relatives. Clear communication and expectation management are essential to build trust and transparency (Bak et al., 2023; Gille et al., 2021).

Importantly, the difficulties in organizing exchange and collaboration experienced by iPSC researchers show that ambiguity in consent forms does not only affect participants’ understanding but also constrains research practice. It is important to note that consent for participating in iPSC research does not necessarily provide a lawful basis for data processing under the GDPR, meaning that an additional data processing consent may be required or other governance structures should be in place to justify the use (Chen and Dove, 2020). Unclear or restrictive consent conditions can prevent iPSC researchers from transferring cell lines, thereby hindering (international) collaboration and validation. This is particularly problematic because (1) resources in the field of rare diseases are already scarce, and (2) exchange of cell lines between academic and commercial parties may be required for drug screening and therapy development (Minssen and Schovsbo, 2014). Ultimately, such constraints can also impede clinical translation. Rather than viewing consent merely as a means to protect the interests and autonomy of participants, these findings support the view that transparent and future-oriented consent procedures also function as a form of research infrastructure (Isasi et al., 2024): they enable collaboration, validation, and downstream application, thereby supporting the broader clinical and societal impact that iPSC-based drug repurposing seeks to achieve.

### Organizing exchange and collaboration: The need for multi-stakeholder alignment

To facilitate international exchange and collaboration, local governance procedures should be streamlined in accordance with international guidelines. For example, interview participants mentioned difficult procedures surrounding the characterization, registration, and transfer of cell lines. Challenges regarding the registration of cell lines have been described previously, for example, in the Australian context (Hu et al., 2024). This is problematic for the reliability and validity of iPSC research results and may have even greater implications for drug repurposing as there might be direct patient implications regarding the effectivity of certain drugs. Increased awareness of international guidelines such as those of the ISSCR can contribute to a smooth transition to clinical trials (International Society for Stem Cell Research, 2025). Researchers should also be encouraged to share and publish their protocols, as well as consent and information templates. It can also be helpful to standardize MTAs for the international exchange of cell lines in collaboration with legal and ethical experts (Chalmers et al., 2014; Lewis and Holm, 2024).

An often underexposed actor in this governance landscape is the local institution that hosts the biobank or laboratory, which typically retains formal control over iPSC lines. These institutions play a pivotal role in translating ethical principles, consent conditions, and regulatory requirements into concrete operational rules. Decisions about access, transfer, characterization, and long-term storage are frequently made at this level, yet they are rarely embedded in broader, participatory governance structures. The challenges regarding the transfer of cell lines suggest that uncertainty at the institutional level—about what is permitted, required, or advisable—can lead to overly cautious practices, such as restricting exchange by default, thereby slowing collaboration and innovation. Interview participants described a strict division of tasks between clinicians and researchers and a dependence on the expertise of other stakeholders. Local institutes therefore bear a critical responsibility to develop clear internal policies, align legal, ethical, and scientific perspectives, and actively facilitate dialogue between researchers, clinicians, legal experts, and patient representatives. A shared sense of urgency to collaborate between the various stakeholders involved in iPSC research is essential. Embedding institutional responsibilities within wider, multi-stakeholder governance frameworks can help ensure that institutional “ownership” of iPSC lines does not become a bottleneck but instead functions as a catalyst for responsible and effective international collaboration.

### Patient engagement: Toward dynamic, transparent, and participatory governance of iPSC research

Patients must be at the center of developing governance structures to ensure that their needs and preferences are safeguarded in iPSC research (Boers et al., 2018; Lensink et al., 2021). The active roles that patient organizations play in the development and maintenance of registries and biobanks are inspiring examples of how to organize such platforms (López et al., 2016). The patient engagement activities mentioned by interview participants show that patient involvement in the governance of iPSC research occurs sporadically but is not structurally embedded and often remains limited to one-way communication rather than a participatory approach (Teare et al., 2015).

To build participatory governance structures, drug repurposing projects can learn from dynamic governance initiatives such as the CHRIS study in Italy and the iPSC biobank in Norway (Isasi et al., 2024; Mascalzoni et al., 2022). These examples demonstrate how active partnerships and shared decision-making can safeguard autonomy and increase transparency in iPSC research (Kaye et al., 2018). It also contributes to the value criterion by continuously enabling participants to re-assess whether new research applications align with their values. A practical solution that could contribute to this ongoing engagement is a built-in system in iPSC biobanks that automatically alerts researchers when patients reach a legally competent age and reconsent might be desirable. The possibility for reconsent could also help to resolve the debate over specific versus broad consent, as it would no longer be necessary to include information on all possible future projects in a single consent form. However, studies also report logistical hurdles in the organization of such platforms (Isasi et al., 2024; Prictor et al., 2018), which may be even more complex for international collaborations. It should also be assured that research participants have sufficient information and understanding to make consent decisions in dynamic platforms, a concern addressed by Mikkelsen et al. (2019). Furthermore, dynamic approaches require a cultural shift for healthcare professionals, researchers, and patients, such as the establishment of new types of collaborations. Ongoing dialogue between researchers and public stakeholders is needed to facilitate mutual exchange and learning over time and to prepare for future uses of iPSCs (Ankeny et al., 2022). As suggested by interview participants, funding bodies can promote the development of effective governance structures for iPSC research with a patient-centered approach from the start.

### Recommendations

Based on the challenges and opportunities addressed by experts in this interview study, we propose eight recommendations that demonstrate how increased stakeholder interaction can improve consent procedures, international exchange and collaboration, and patient engagement in the governance of iPSC-based drug repurposing for rare diseases, which may also be relevant for other iPSC research contexts (Figure 1).

**Figure 1:**
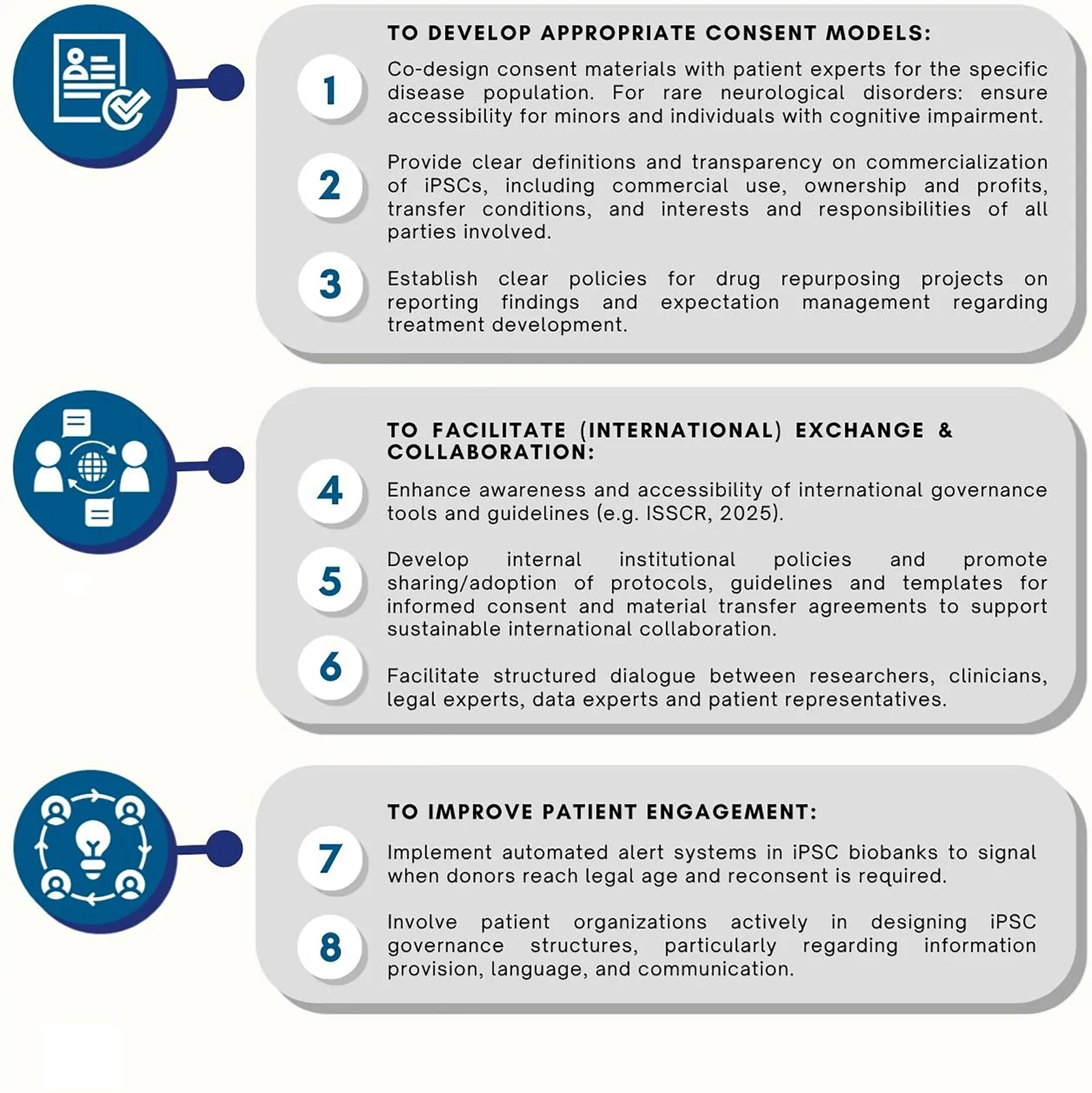
Recommendations for the governance of iPSC-based drug repurposing for rare diseases

### Strengths and limitations

This study provides insight into the current practice of iPSC-based drug repurposing by exploring the perspectives of a variety of experts from different countries based on a real-world international research context. This multi-stakeholder perspective provides a broad overview of the challenges and opportunities and allows for the development of recommendations that can be implemented in practice. However, as our sample of interview participants is recruited based on a specific research context of the SIMPATHIC consortium, our findings do not represent a complete overview of all the challenges and opportunities experienced in iPSC-based drug repurposing. The views of the interview participants described in this study cannot be generalized to all experts, countries, and iPSC research contexts. For example, this study does not include the perspectives of commercial parties conducting research with iPSCs. The consent criteria of Mikkelsen et al. (2019) and Barnes et al. (2025) proved to be a useful framework for categorizing and evaluating current consent conditions in safeguarding ethical principles in iPSC research, while at the same time enabling us to identify the limitations of governance structures in meeting the needs of all stakeholders involved. These shortcomings can only be identified by integrating the perspectives of the different experts involved, with a central role for patient experts. It would be even more effective to facilitate this process through multi-stakeholder dialogues. In addition to the experts included in this study, the recommendations should be critically evaluated by policymakers and regulators to assess their feasibility and applicability in existing local governance structures.

### Conclusion

This study examined current practices, challenges, and opportunities in the governance of iPSC research, with a focus on iPSC-based drug repurposing for rare neurological disorders. This study demonstrates the relevance of specifying criteria for consent in a specific research context. At the same time, the results show that these criteria are not sufficient to meet the needs of all stakeholders involved. Consent procedures should be tailored to the needs and preferences of patients. Furthermore, multi-stakeholder alignment is identified as a key aspect to facilitate international exchange and collaboration. Developing participatory governance structures with a patient-centered approach is essential for responsible research in iPSC-based drug repurposing. Other iPSC research contexts can adopt a similar approach to co-create transparent and dynamic governance structures that can respond quickly to the challenges and opportunities in the field.

## Resource availability

### Lead contact

Requests for further information and resources should be directed to the corresponding author, Esther A. M. Bührman (e.a.m.buhrman@amsterdamumc.nl).

### Materials availability

No materials were generated in this study.

### Data and code availability

The original transcripts of the interview data reported in this study are not publicly available due to privacy considerations in line with the data management policy of the Amsterdam UMC. The interview code system has been attached in supplemental information S4.

## Acknowledgments

We would like to thank all interview participants for their time and contribution to this study, as well as all members of the SIMPATHIC consortium who participated in discussions on the governance of iPSC research. We also appreciate the comments from two anonymous reviewers, which helped improve the manuscript. This project has received funding from the 10.13039/100018693Horizon Europe research and innovation program under grant agreement no. 101080249.

## Author contributions

Conceptualization, E.A.M.B., M.C.C., W.P.C.B., and T.R.; methodology, all authors; investigation, E.A.M.B.; data curation, E.A.M.B.; formal analysis, E.A.M.B.; validation, E.A.M.B., A.G., V.M.H., and T.R.; writing – original draft, E.A.M.B.; writing – review and editing, all authors; visualization, E.A.M.B.; supervision, M.C.C., W.P.C.B., and T.R.; project administration, M.C.C. and T.R. All authors have read and agreed to the published version of the manuscript.

## Declaration of interests

The authors declare no competing interests.

## STAR★Methods

### Experimental model and study participant details

#### Research design

This study used a qualitative, interview-based research design to provide in-depth insights into the challenges and opportunities that stakeholders perceive in iPSC-based drug repurposing. Qualitative research allows for a deeper understanding of personal perspectives, values, and interests. It also provides insight into the real-life research practice and informal governance mechanisms.

#### Participant sampling and recruitment

Semi-structured interviews with stakeholders were conducted. Purposive sampling was used to approach iPSC researchers in the field of (rare) neurological disorders and other professionals involved in the governance of iPSC research. Relevant participants were identified through the network of the SIMPATHIC consortium, recent publications related to the governance of iPSC research, and referrals from (potential) interview participants. The interview participants had to be either iPSC researchers or clinicians in the field of (rare) neurological disorders or other experts involved in the governance of iPSC research, such as patient experts, ethical experts and/or legal experts. Data experts managing iPSC biobanks and/or registries and commercial parties conducting research with iPSCs were also approached. Most of the interview participants were located in European countries, because 1) the context of the SIMPATHIC project was taken as a starting point for this study, with most of the iPSC-laboratory work being carried out in the Netherlands and Germany, and 2) we aimed to explore governance challenges in a context where international collaboration takes place within a largely shared legal framework under the GDPR. One Australian expert was also included to compare and learn from other iPSC governance initiatives.

### Method details

#### Interview guide

An interview guide (see supplemental information S2) was compiled based on the ethical and regulatory challenges identified in literature: the topics were for a large part based on two interview studies on organoid biobanking for cystic fibrosis (Lensink et al., 2021; Lensink et al., 2020). The interview guide was co-developed with a patient expert [A.G.] and an iPSC researcher [V.M.H] and further validated after a pilot interview.

#### Interview procedure and analysis

The interviews took approximately 60 min and were held in person or through an online video call via Microsoft Teams in case of practical constraints. During the interviews, the researcher took notes of observations and important information. A summary with bullet points was created directly after the interview. All interviews were recorded and transcribed by the researcher, after which the audio-files were deleted. A thematic analysis of the transcripts was performed in MAXQDA 24, using a combination of deductive coding (following the interview guide topics) and inductive coding for emerging themes (such as ethical considerations and values). During the analysis, the themes were continuously evaluated. After six interviews, a preliminary codebook was discussed with a co-author [TR] and consensus was reached on a preliminary coding structure. We observed thematic saturation at the level of the overall dataset, with no new governance themes emerging after 14 interviews (see supplemental information S4), suggesting sufficient thematic saturation for the purposes of answering our research question. We then conducted three more interviews which confirmed thematic saturation, after which data collection was concluded. The final code system can be found in supplemental information S3.

#### Ethics and data management

The Medical Ethics Review Committee of Amsterdam University Medical Centers evaluated the study design and decided that the Medical Research Involving Medical Subjects Act (WMO) does not apply to this study and that further official approval was not required (2024.0399).

All interview participants signed informed consent prior to the interview. They were explicitly asked for permission to record the interview and informed about the use and storage of their data in accordance with the data management plan established in Amsterdam UMC. The transcripts were pseudonymized by using codes and removing personal information. We did not collect data on the sex, gender, ancestry, race, ethnicity, socioeconomic status, and age of the study participants, as these factors are not relevant to answering our research question.

### Quantification and statistical analysis

No statistical analysis was performed. Quantification was not applicable to this qualitative study.

### Additional resources

No additional resources were created.

## Supplemental information

Document S1. SIMPATHIC consortium collaborators

Document S2. Interview guide

Document S3. Interview code system

Document S4. Number of new codes per interview

Document S5. Footnotes
