# Supplementary material for: From consent to participatory governance: The need for multi-stakeholder alignment in iPSC-based drug repurposing
Source: Stem Cell Reports. 2026 Sep 3;21(10):103062. doi: 10.1016/j.stemcr.2026.103062 (PMC13681317; doi:10.1016/j.stemcr.2026.103062)
Supplement: Supplementary file 1 [file mmc2.pdf]

# Supplemental information Document S1. Interview guide

**Introduction**

Thank you for participating in this interview on the governance of iPSC research. I would like to ask you to share your experience and perspectives on iPSC research, including topics as consent procedures, international exchange and collaboration, and patient engagement. I am curious to learn what challenges and opportunities you see for iPSC-based drug repurposing, specifically in the context of rare neurological disorders. Please let me know if anything is unclear or if you have additional remarks during the interview.

**Background / Experience**

*iPSC research*

- How are you involved in iPSC research?
- What kind of iPSC models are you working with?

*Local procedures*

- Are there specific guidelines or SOPs for working with iPSC models within your organization?
    - o What are these guidelines based on?
    - o To what extent do these match with the ISSCR?
- Has the regulation for your iPSC research changed over time? How?

**Consent**

*Consent procedure*

- When are patients approached for iPSC research?
- How is the information about iPSC research provided?
    - o Who provides the information?
    - o What kind of information is provided?
        - Is specific consent required for the generation of iPSCs?
- How is informed consent obtained? How is consent given?
    - o Is oral consent sufficient, or is written consent required?
    - o Is the consent given on paper or online?
    - o Is a signature required?
- Where is consent registered? Who is responsible for this?

*Non-competent individuals*

- How is consent obtained from individuals that are legally incompetent?
    - o Children (with rare neurological disorder)
    - o Patients with cognitive impairment

*Re-consent*

- Is consent renewed after a certain time? If yes:
    - o Who is responsible for updating the consent?
    - o (How) has this process been automated?
- Do you think reconsent is necessary in iPSC research? Why?
- What would for you be reasons to ask for reconsent? F*or example:*
    - o *Because of age*
    - o *Because of a new study*
    - o *Because of scientific/technological developments*

*Consent form*

- How are the consent forms generated?
    - o Who is responsible for this?

    - Is the consent form checked by an ethical review committee?
- Does the [hospital/lab/company] use standardized consent forms?
  (Or harmonized on another level? *EU/professional organizations*)
- Is the consent form based on certain guidelines or laws & regulations?

*Consent topics*

- Are there different options that patients can give consent for? Why (not)?
  *For example: transfer to another institution, company or country.*

**Ethical use**

*Ethical review*

- What does the ethical review procedure of iPSC research look like?
- Is there a special procedure or committee for iPSC research?
  *(local IRB, national, etc.)*
- What (ethical) considerations are taken into account?
  *For example: privacy, autonomy, control*
- Who are involved in the ethical review?
- Are there other parties consulted?
    - *For example: legal experts, data experts*
    - Is there a role for patients/patient organizations?

*Purpose*

- For what purposes can iPSCs be used?
- Are there different considerations for different types of models?
  *(brain-on-chip vs 2D co-cultures vs organoids)*
- Are there different considerations for neuronal stem cell models?

**International exchange and collaboration**

*Storage*

- Where are the iPSCs stored?
- How long can the iPSCs be stored? What is this period based on?
- Who owns/manages the iPSCs? Is there a special committee for this?

*Registration*

- Do you use a [local/national/international] database for iPSC data registration?
- Which information is registered?

*Transfer*

- Do you have any experience with transferring patient material? If yes:
    - What are your experiences with the MTA procedure?
    - How do you maintain terms & conditions?
      *(management / ownership / ethical use)*
    - Who is involved in this process?

*Harmonization*

- Are you aware of any differences in regulation between countries/organizations?
- Have these differences ever influenced/limited the possibilities for iPSC research/collaboration?
- How can regulation be harmonized?

**Patient engagement**

*Communication/Transparency*

- How can patients see what research has been performed?
- How are they informed about findings/discoveries?
- Are publications open access available?

*Involvement in research*

- How are patients *(individually/group)* currently involved in iPSC research?
- Are patient preferences considered in the design of the research?
- Are patients / patient organizations involved in the design of consent forms?
- What is needed for patient engagement in iPSC-based drug repurposing?

**Closing questions**

- Do you see other challenges or opportunities in the governance of iPSC-based drug repurposing? *(Specifically for rare neurological disorders)*
- What are according to you conditions for responsible use of iPSCs for drug repurposing?
