# Supplementary material for: From consent to participatory governance: The need for multi-stakeholder alignment in iPSC-based drug repurposing
Source: Stem Cell Reports. 2026 Sep 3;21(10):103062. doi: 10.1016/j.stemcr.2026.103062 (PMC13681317; doi:10.1016/j.stemcr.2026.103062)
Supplement: Supplementary file 2 [file mmc4.pdf]

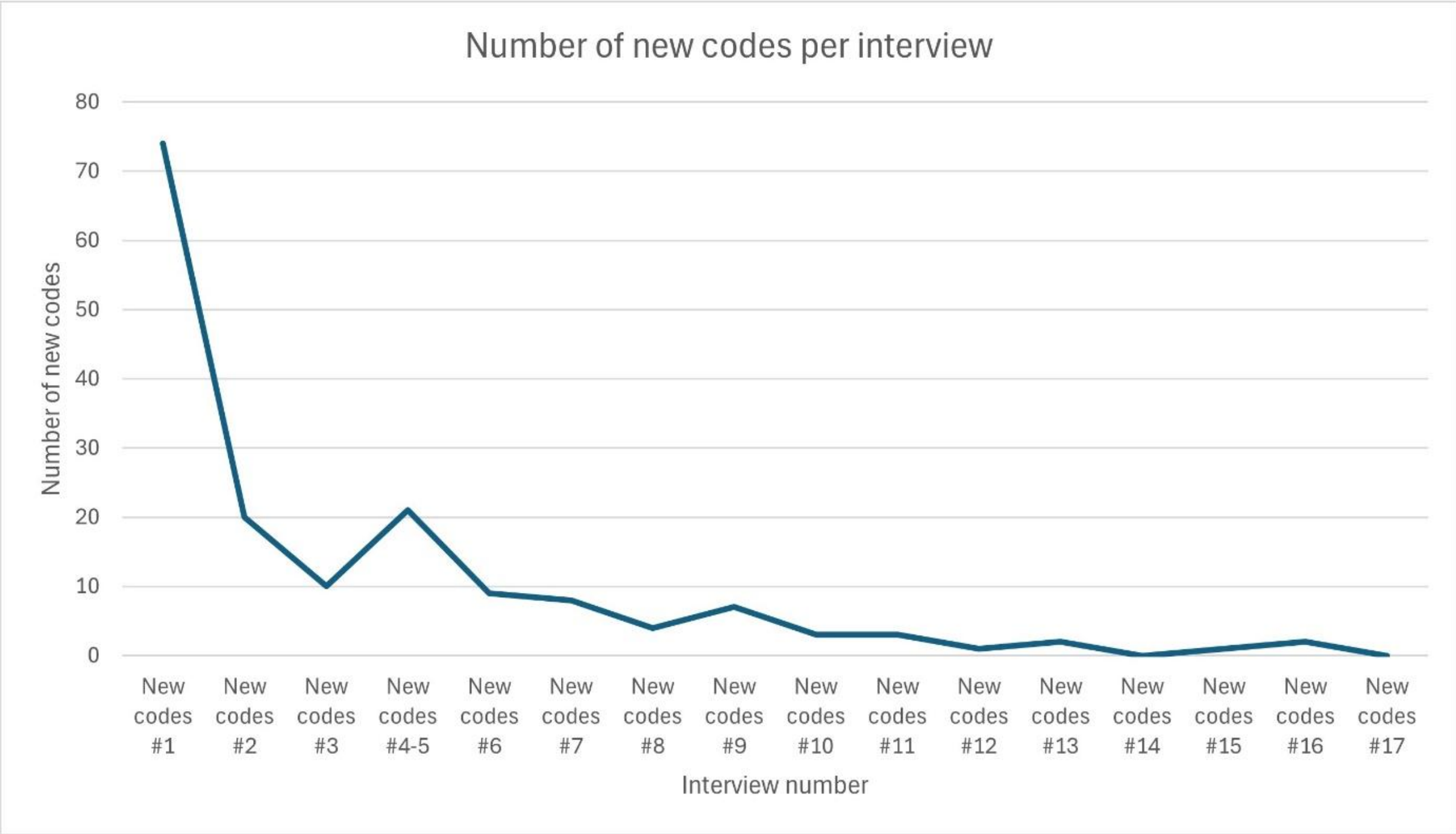

Supplemental Document S4. The number of new codes per interview. The X-axis shows the interview number, the Y-axis shows the number of new codes. The trend line suggests that data saturation was reached after approximately 14 interviews.
