# Supplementary material for: From consent to participatory governance: The need for multi-stakeholder alignment in iPSC-based drug repurposing
Source: Stem Cell Reports. 2026 Sep 3;21(10):103062. doi: 10.1016/j.stemcr.2026.103062 (PMC13681317; doi:10.1016/j.stemcr.2026.103062)
Supplement: Supplementary file 3 [file mmc5.pdf]

Supplemental information Document S5. Footnotes

i. In addition to *formal* laws and regulations, it is essential to take into account *informal* governance structures, as they largely determine internal and local procedures, and differences in these procedures may hinder collaboration in iPSC research. Furthermore, innovative research methods and projects often rely on *informal* governance when *formal* legislation is still under development, which is the case for iPSC research.

ii. Lensink et al. (2022), among others, argue that ‘participant’ is a more appropriate term when referring to people who provide human tissue for research, as this term recognizes the ongoing relationship between the tissue provider and the tissue user. In this study, we have decided to use the word ‘patient’ to prevent confusion with other stakeholders in iPSC research and to make an active distinction between patient participants and other participants in iPSC research, who may also be non-patients, parents of patients, etc. We have furthermore avoided to use the word ‘donor’ as much as possible.

iii. We would like to emphasize that ‘engagement’ involves more than ‘participation’. In line with Teare et al. (2015), we define engagement as a two-way interaction between participants and researchers, with an active role of the biobank to provide regular updates and information and possibilities for reconsent.
